# Clinical and genetic characteristics of two patients with tyrosinemia type 1 in Slovenia – A novel fumarylacetoacetate hydrolase (*FAH*) intronic disease-causing variant

**DOI:** 10.1016/j.ymgmr.2021.100836

**Published:** 2021-12-16

**Authors:** Jaka Sikonja, Jernej Brecelj, Mojca Zerjav Tansek, Barbka Repic Lampret, Ana Drole Torkar, Simona Klemencic, Neza Lipovec, Valentina Stefanova Kralj, Sara Bertok, Jernej Kovac, Barbara Faganel Kotnik, Marketa Tesarova, Ziga Iztok Remec, Marusa Debeljak, Tadej Battelino, Urh Groselj

**Affiliations:** a Faculty of Medicine, University of Ljubljana, Ljubljana, Slovenia; b Department of Gastroenterology, Hepatology and Nutrition, University Children's Hospital, UMC Ljubljana, Ljubljana, Slovenia; c Department of Endocrinology, Diabetes, and Metabolic Diseases, University Children's Hospital, UMC Ljubljana, Ljubljana, Slovenia; d Clinical Institute for Special Laboratory Diagnostics, University Children's Hospital, UMC Ljubljana, Ljubljana, Slovenia; e Unit for Clinical Dietetics, University Children's Hospital, UMC Ljubljana, Ljubljana, Slovenia; f Department of Haematology and Oncology, University Children's Hospital, UMC Ljubljana, Ljubljana, Slovenia; g Department of Pediatrics and Adolescent Medicine, First Faculty of Medicine, Charles University and General University Hospital in Prague, Prague, Czech Republic

**Keywords:** Tyrosinemia, Fumarylacetoacetate hydrolase, Nitisinone, Dried blood spot, Succinylacetone, Intronic variant, AFP, alpha-fetoprotein, ALP, alkaline phosphatase, ALT, alanine transaminase, AST, aspartate transaminase, DBS, dried blood spot, FAH, fumarylacetoacetate hydrolase, GGT, gamma glutamyl transferase, HT1, tyrosinemia type 1, INR, international normalized ratio, MS/MS, tandem mass spectrometry, NBS, newborn screening, NTBC, nitisinone, PTT, partial thromboplastin time, RF, reference range, SA, succinylacetone

## Abstract

Tyrosinemia type 1 (HT1) is an inborn error of tyrosine catabolism that leads to severe liver, kidney, and neurological dysfunction. Newborn screening (NBS) can enable a timely diagnosis and early initiation of treatment.

We presented the follow up of the only two Slovenian patients diagnosed with HT1. Metabolic control was monitored by measuring tyrosine, phenylalanine and succinylacetone from dried blood spots (DBSs). Retrograde screening of HT1 was performed from DBSs taken at birth using tandem mass spectrometry.

First patient was diagnosed at the age of 6 months in the asymptomatic phase due to an abnormal liver echogenicity, the other presented at 2.5 months with an acute liver failure and needed a liver transplantation. The first was a compound heterozygote for a novel *FAH* intronic variant c.607-21A>G and c.192G>T whereas the second was homozygous for c.192G>T. At the non-transplanted patient, 66% of tyrosine and 79% of phenylalanine measurements were in strict reference ranges of 200–400 μmol/L and >30 μmol/L, respectively, which resulted in a favorable cognitive outcome at 3.6 years. On retrograde screening, both patients had elevated SA levels; on the other hand, tyrosine was elevated only at one.

We showed that non-coding regions should be analyzed when clinical and biochemical markers are characteristic of HT1. DBSs represent a convenient sample type for frequent amino acid monitoring. Retrograde diagnosis of HT1 was possible after more than three years of birth with SA as a primary marker, complemented by tyrosine.

## Highlights

• Non-coding region variants of *FAH* gene can result in a symptomatic HT1.
• Retrograde screening for HT1 is technically possible even three years after birth.
• DBS are convenient for monitoring HT1 patients and are family-friendly.
• Regular monitoring in HT1 patients can result in a favorable cognitive outcome.

## 1 Introduction

Tyrosinemia type 1 (HT1; OMIM 276700) is a rare autosomal recessive metabolic disorder caused by a defect in fumarylacetoacetate hydrolase (FAH), the last enzyme in the tyrosine catabolic pathway. Insufficient activity of FAH leads to the accumulation of toxic metabolites such as succinylacetone (SA). These metabolites disrupt the cellular metabolism in various body tissues, predominantly in liver, kidneys, and central nervous system [1]. Incidence of HT1 is estimated at one case per 100,000 [2].

Age at onset of symptoms correlates to the severity of HT1. Poor prognosis and a severe disease course are associated with an earlier manifestation of symptoms, however either an acute or chronic form of HT1 can present with liver disease at any age. The spectrum of liver disease ranges from an acute liver failure to hepatic cirrhosis and hepatocellular carcinoma. In kidneys, proximal tubular defect results in Fanconi syndrome and subsequent development of rickets. Nervous system impairment manifests with episodes of peripheral neuropathy. Untreated HT1 causes death at a young age [3], [4], [5].

Nitisinone (NTBC) discovery represents an important milestone in the treatment of HT1. Before its introduction, patients needed to adhere to a diet until a liver transplantation could be performed. NTBC blocks 4-hydroxyphenylpyruvate dioxygenase which reduces the level of toxic metabolites and increases tyrosine concentration. Protein-restricted diet itself is not therapeutic for HT1 and should complement the therapeutic effect of NTBC to prevent the complications of hypertyrosinemia. Nowadays, liver transplantation at HT1 patients is indicated at a liver failure in an undiagnosed patient, at a NTBC treatment failure and at a hepatocellular carcinoma emergence [6], [7], [8].

Newborn screening (NBS) from dried blood spots (DBSs) enables a timely diagnosis and an early NTBC initiation which can prevent liver dysfunction [9], [10], [11]. The main goal of treatment is the suppression of SA concentration as it is considered a surrogate parameter of toxicity. Additionally, monitoring should include regular measurements of NTBC, phenylalanine and tyrosine concentrations [11], [12], [13], [14].

In the following paper, we present clinical and genetic characteristics of the only two Slovenian patients, diagnosed with HT1, results of a retrograde screening for HT1 and a novel intronic disease-causing variant in the *FAH* gene.

## 2 Case presentations

### 2.1 Case 1

Patient 1 was referred to the University Children's Hospital in Ljubljana for further investigations of abnormal liver echographic findings at the age of 5.5 months. Family history was positive for maternal hypothyroidism and hyperlipidemia. Patient 1 was born at term with a birth weight 3520 g and a birth length 54 cm after an induction of vaginal delivery due to maternal hypertension. Pregnancy was otherwise uneventful. In the neonatal period, he had elevated bilirubin levels but did not need any intervention. He was feed with a combination of mother's milk and milk formulas. Parents just started introducing complementary food prior to presentation. At 3 months of age, he suffered from a urinary tract infection and its evaluation revealed hyperechogenic liver on ultrasound. Next liver sonography at 5.5 months of age showed enlarged liver with nonhomogeneous parenchyma and hypoechogenic lesions, but without the presence of free fluid in the peritoneal cavity. Throughout infancy he had no symptoms of liver disease and initial laboratory evaluation was suggestive of a liver function impairment. Aspartate transaminase (AST) activity was increased (102 U/L; reference range (RF): < 51.6 U/L), whereby alanine transaminase (ALT), a more specific liver enzyme, was just above the normal range (38.4 U/L; RF: < 36 U/L). Gamma glutamyl transferase (GGT) (278 U/L; RF: 1.2–39 U/L), alkaline phosphatase (ALP) (1061 U/L; RF: 82–384 U/L) and bilirubin (1.5 mg/dL; RF: < 1.0 mg/dL) were elevated. Despite slightly raised ammonia (59.9 μmol/L; RF: 21–50 μmol/L) he did not have signs of hepatic encephalopathy. Prolonged partial thromboplastin time (PTT) (56.9 s; RF: 35.1–46.3 s) and increased international normalized ratio (INR) (1.6; RF: 0.86–1.22) were not accompanied by a history of bleeding. Albumin levels were normal (40 g/L; RF: 32–55 g/L). Phosphorous concentration was just above the lower limit of normal for his age (4.96 mg/dL; RF: 4.6–8.0 mg/dL).

In metabolic workup, DBS SA was persistently elevated and ranged from 3.47 to 4.43 μmol/L (RF: <1 μmol/L) prior to NTBC initiation. Alpha-fetoprotein (AFP) concentrations were above 20.000 ng/mL (RF: 1.25–129 ng/mL [15]). Next-Generation Sequencing was performed using MiSeq desktop sequencer coupled with MiSeq Reagent kit v3 (both Illumina, San Diego, USA). The regions of interest were enriched using TruSight One library enrichment kit (Illumina, San Diego, USA) following manufacturer's instructions. A panel of genes causing tyrosinemia was analyzed (*FAH*, *GSTZ1*, *HPD*, *TAT*). Possibly causative variants were confirmed by targeted Sanger sequencing using custom oligonucleotides, BigDye Terminator v3.1 sequencing kit and ABI Genetic Analyzer 3500 (both Applied Biosystems, USA). Despite high clinical suspicion of HT1, initial genetic evaluation did not confirm the diagnosis. A heterozygous single nucleotide substitution NM_000137.4:c.192G>T in *FAH* gene inherited from his mother was identified. Therefore, his medical state could not be explained by a single disease-causing variant because variants in *FAH* gene are inherited in an autosomal recessive pattern. Additional whole genome sequencing revealed an intronic substitution NM_000137.4:c.607-21A>G inherited from his father. Retrograde analysis of DBS taken at birth was performed at the age of 3.1 years by tandem mass spectrometry (MS/MS): SA and tyrosine concentrations were 1.31 μmol/L and 364 μmol/L (RF for NBS: 0–0.30 μmol/L and 0–320 μmol/L, respectively).

At 6 months of age, diagnosis of HT1 was based on elevated SA in DBS, abnormal liver tests and a single disease-causing variant in *FAH* gene. Immediately, the boy was started on NTBC and a phenylalanine/tyrosine-restricted diet. His parents observed a self-limiting diarrhea in the first two weeks after treatment initiation. Due to the nature of treatment type, amino acid status was frequently monitored in DBSs and plasma. Methods used for retrograde and prospective analysis of plasma and DBS samples were described by Smon et al. [16]. Boy's parents received instructions to send a filter paper with a DBS every 1–2 weeks that was analyzed for phenylalanine and tyrosine. Extended evaluation of plasma amino acids, DBS SA and liver tests was done at routine out- and in-patient visits. Parental compliance with the treatment and monitoring regime resulted in more than 160 measurements of phenylalanine and tyrosine throughout the follow up period. The average time between DBS samples was around 8 days.

**Fig. 1:**
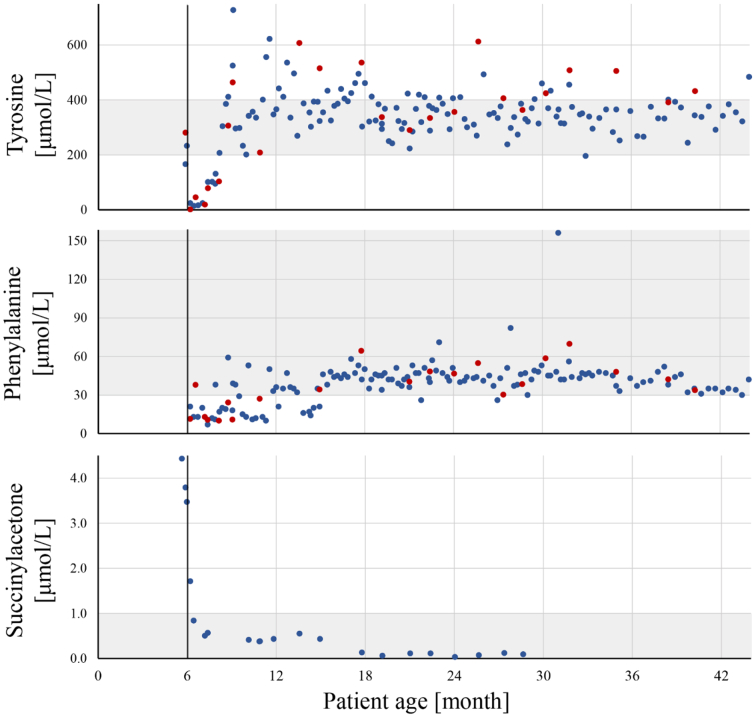
Tyrosine and phenylalanine concentrations in dried blood spots and plasma during follow up in patient 1. Start of treatment is illustrated with a vertical line at 6 months of age. Target range 200–400 μmol/L for tyrosine, >30 μmol/L for phenylalanine and <1 μmol/L for succinylacetone are presented in gray. Blue dot – tandem mass spectrometry analysis of dried blood spots, red dot – plasma analysis by amino acid analyzer.

**Fig. 2:**
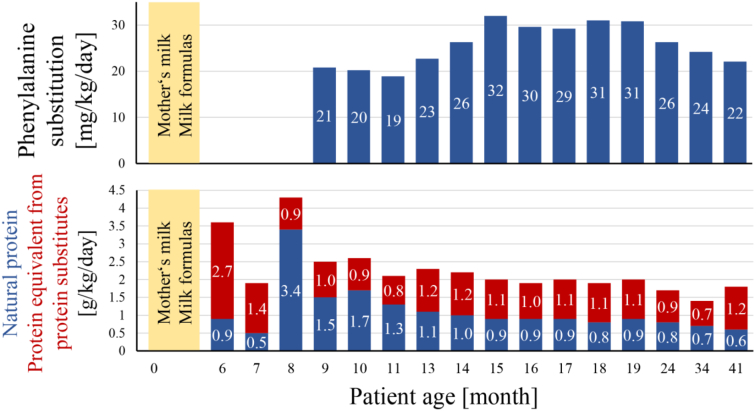
Average total daily protein intake of natural protein, protein equivalent from protein substitutes free from phenylalanine and tyrosine and phenylalanine substitution derived from dietary intake analysis in patient 1. Note: Values on the horizontal axis are not proportionately dispersed.

At diagnosis, he was introduced 1.23 mg/kg/day of NTBC in two daily doses which decreased due to his growth and reached a minimum of 0.57 mg/kg/day. Additionally, a clinical dietitian helped the parents with meal planning to ensure that appropriate amount of natural protein and protein substitute were received. Fig. 2 shows the adjustments of dietary preparations. For the monitoring of amino acid concentrations, we strived to keep the values in the target range 200–400 μmol/L for tyrosine and > 30 μmol/L for phenylalanine and managed to have 66% and 79% of values through the whole follow up in range, respectively. Optimal metabolic control (shown in detail in Table 1 and graphically presented in Fig. 1) was achieved at 20 months of age as 75% and 98% of tyrosine and phenylalanine measurements were in range in the later period and normalization of liver function tests, AFP and SA was observed.

**Table 1:** Overview of metabolic control at patient 1.

	Tyrosine			Phenylalanine		
Target range [μmol/L]	200–400			> 30		
Sample type	DBS	Plasma	Total	DBS	Plasma	Total
Number of samples	141	24	165	139	21	160
Follow up period – in range [%]	71	38	66	81	67	79
Patient age at achievement of optimal control [month]	20			15		
Prior period – in range [%]	57	31	53	38	22	35
Later period – in range [%]	82	45	78	98	100	98

Abbreviations: DBS – dried blood spot.

**Fig. 3:**
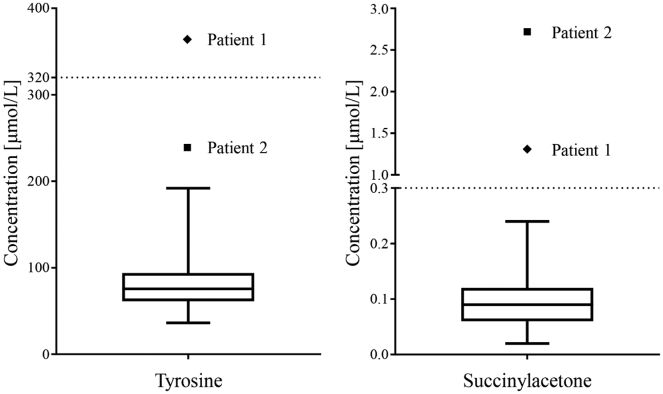
Comparison of tyrosine and succinylacetone concentrations between healthy newborns and patients with HT1. Values from healthy controls are presented with a box and whiskers plot. The box represents the 25th and 75th percentiles, the line in the box represents the median, and the whiskers are the 1st and 99th percentiles. Succinylacetone and tyrosine concentrations were determined from dried blood spots using tandem mass spectrometry. Patient samples were analyses retrograde. Cut-off values for a positive screening result are presented with dotted lines.

Kidney function remained stable at patient 1, however decreased bone density was measured by x-ray absorptiometry (Z scores −2.4 and − 1.7). Neither corneal crystals and hepatocellular carcinoma were detected on routine physical and imaging examinations. High general and verbal intellectual ability and a normal neurocognitive development was noted at the age of 3 year 7 months [17], [18], [19], [20], [21].

### 2.2 Case 2

Patient 2 was referred to our hospital at 2 months of age after three days of persistent fever, diarrhea and vomiting that progressed to the clinical presentation of acute abdomen. Family history was unremarkable. Gestational diabetes was noted during pregnancy and was well controlled. The boy was born at term with a birth weight 3450 g and a birth length 51 cm. The neonatal period was complicated by mild icterus; no hypoglycemia and hypotonia were observed. For the first two weeks, he was breastfed but was later given milk formulas due to poor suction. Exploratory laparotomy of acute abdomen revealed only the presence of ascites. Serum transaminases at presentation were barely elevated (ALT 50.4 U/L, RF: < 36 U/L; AST 59.4, RF: < 51.6 U/L) and at the same time heavy liver function impairment was evident from low albumin levels (20 g/L; RF: 32–55 g/L) and disruptive coagulation (INR 9.47, RF: 0.86–1.22; PTT > 160 s, RF: 35.1–46.3 s; Fibrinogen 0.6 g/L, RF: 0.8–3.83 g/L). Initially, ammonium levels were normal but with the progression of liver disease rose to 330 μmol/L and had to be corrected by hemodialysis. NTBC was started at a dose of 1.0 mg/kg/day, however patient 2 still required intensive care due to severe cerebral edema, hemodynamic instability, electrolyte imbalance and bleeding. At 4 months of age, liver transplantation was performed at the Pediatric liver transplantation center in Bergamo. A comparison between patients' main characteristics is presented in Table 2.

**Table 2:** Characteristics of patients at first presentation and during follow up period.

	Patient 1	Patient 2
**General**		
Sex	Male	Male
Identification with retrograde newborn screening	Yes	Yes
Genotype – *FAH* gene	Heterozygote, c.192G>T and c.607-21A>G	Homozygote, c.192G>T

**First presentation**		
Clinical features at presentation	3 months: liver hyperechogenicity, without clinical symptoms 5.5 months: abnormal liver tests, ultrasound shows enlarged liver, nonhomogeneous parenchyma with hypoechogenic lesions, without ascites, normal kidney function, neurological status appropriate for age	2 months: acute liver failure with ascites, bleeding and hepatic encephalopathy, acute renal injury
Age at diagnosis	6 months	2.5 months

**Follow up period**		
Treatment type	Nitisinone and diet	Liver transplantation at 4 months
Growth	Normal	Normal
Bone density	Lowered	Normal
Neurological and psychiatric state	Normal development	Lowered muscle tone, moderate cognitive impairment, impaired speech development, short attention span, hyperactivity
Other HT1 related comorbidities	None	Hypertrophy of interventricular septum
Treatment complications	Diarrhea	CMV and EBV reactivation
Age of patient at last follow up	3 years 8 months	6 year 2 months

Patient 2 had a homozygous nucleotide change NM_000137.4:c.192 > T in the *FAH* gene. Genetic analysis was performed as at patient 1. Retrograde analysis of DBS taken at birth was performed 5.2 years after DBS collection and it revealed SA and tyrosine concentration of 2.72 μmol/L and 239, respectively.

No signs of ascites and hepatocellular carcinoma were found on regular imaging examinations. Non-progressive left ventricular hypertrophy which was most expressed in the interventricular septum persisted throughout the follow up. Kidney function and vision remained intact. Bone density and body growth were normal.

Neurocognitive development was impaired despite liver transplantation. Patient 2 had extremely low general intellectual ability, including verbal and performance intellectual ability at the age of 5 years and 5 months. Parents reported of a short attention span and hyperactivity. He regularly visited an occupational therapist, a speech therapist, and a special educator to promote his development.

## 3 Discussion

We described two patients suffering from the same metabolic disease but with completely different disease courses. The first presented within the first few months with abnormal liver echogenicity and was started on NTBC and protein-restricted diet. The second patient presented in the same period with an acute liver failure which could not be reversed by NTBC and he had to undergo a curative liver transplantation.

Patient 1 was diagnosed in a clinically asymptomatic period. His liver showed an abnormal parenchymal echogenicity and his laboratory results revealed significant elevation of ALP and AFP which is in accordance with previous reports [22], [23]. Moreover, HT1 was spontaneously discovered due to a HT1-unrelated medical state and would most likely present with symptoms at an older age. On the other hand, patient 2 developed a severe HT1 presentation, marked by an acute liver failure and bleeding tendency that required intensive care [22], [23], [24]. We assume this patient had an irreversible liver damage that could not be corrected by NTBC and was the driving factor of the disease course. Hence, prompt SA measurement to diagnose HT1 should be performed in infants that present with an unexplained liver dysfunction characterized by a combination of a severe disruption of coagulation and non-significant elevations of aminotransferases.

Different clinical presentation in patients is probably due to their different genetics. Patient 2 had a homozygous nucleotide substitution of *FAH* gene c.192G>T, while patient 1 was compound heterozygous for c.192G>T and a novel intronic variant. c.192G>T originates from the Pakistani region [2] and results in an amino acid substitution (p.Gln64His) and also alters the process of intron splicing since it changes the last nucleotide in exon 2. The firstly described intronic region variant c.607-21A>G at patient 1 causes the loss of exon 9 in the mature FAH mRNA due to alternative splicing and probably destabilizes the mRNA molecule. The combination of variants in patient 1 results in low levels of FAH mRNA and low FAH activity.

Homozygosity for c.192G>T in patient 2 is associated with a severe HT1 phenotype [25]. On the other hand, a combination of c.192G>T and a functional *FAH* sequence with an intronic variant resulted in a milder phenotype as seen in patient 1 who did not have clinically significant symptoms up to 5 months of age. Nucleotide variant c.607-21A>G has not yet been described in HT1 patients and is not present in the general population (dbSNP, gnomAD). We can learn an important lesson from patient 1: when clinical and biochemical presentation is characteristic of HT1 and the genetic analysis of the exon regions of *FAH* gene speak against its diagnosis, additional analysis of intronic regions should be performed.

Newborn screening for HT1 is performed in various countries around the world. SA is the primary screening marker due to its specificity and sensitivity while tyrosine is used as a complementary parameter [10], [23]. Diagnosis through NBS and prompt treatment is related to a favorable outcome with less disease complications [23], [24], [26]. Both presented patients were born prior to the nationwide implementation of an expanded NBS in Slovenia and we performed the retrograde analysis to assess the sensitivity of the newly implemented screening program for HT1. Retrograde analysis of DBS taken at birth showed elevated SA levels; on the other hand, tyrosine was elevated just at patient 1. Normal tyrosine levels at NBS are not uncommon and do not exclude HT1 [27]. Both tyrosine and SA levels were above the 99th percentile compared to healthy controls (Fig. 3). SA levels were higher at the patient with an acute liver failure (2.72 μmol/L vs. 1.31 μmol/L) which indicates a possible relationship between SA levels at NBS and the severity of HT1 however, further studies are needed to confirm this statement. From these results, we assume that both patients would have been recognized by NBS and that early diagnosis could have improved their outcome, maybe even prevent an acute liver failure at the second patient.

Persistent hypertrophic cardiomyopathy present at patient 2 was hemodynamically insignificant and predominantly involved the interventricular septum. This is in contrast with the previous reports where a benign hypertrophic cardiomyopathy completely resolved either after a liver transplantation or NTBC treatment [8], [28]. However, Arora et al. described a persisting cardiomyopathy in a patient with a severe HT1 course [29].

NTBC is widely accessible to the majority of patients across Europe [30] and reduces the need for hospital admissions and diminishes the incidence of acute HT1 complications. Moreover, NTBC significantly reduces the costs of hospitalizations, but on the contrary the NTBC therapy itself is related to high costs [31]. To ensure the optimal management of presented children, NTBC and dietary supplements were fully covered by the Slovenian health insurance.

Treatment monitoring at patient 1 was done by regular measurements of tyrosine and phenylalanine in DBSs since DBSs allow home blood collection and can be easily sent over long distances, thereby making them family-friendly [13]. However, plasma represents a gold standard sample type and amino acid concentrations measured in plasma are slightly higher from DBS values. Thus, it is advised that the treating clinicians are aware of the used method for metabolic monitoring [16], [32], [33]. We strived to keep tyrosine concentrations in a strict range of 200–400 μmol/L derived from proposed ranges in the literature [9], [12], [23], [34]. Because phenylalanine is an essential amino acid, low phenylalanine levels can affect skin, growth, and neurological development and the proposed target levels by the experts for phenylalanine are >30 μmol/L [34], [35]. SA should be maintained below 1 μmol/L [9], [23].

Major differences can be seen when comparing neurocognitive functioning of presented patients. At the time of evaluation, patient 1 had high intellectual capabilities with no significant emotional or behavioral problems, while patient 2 had a significant cognitive impairment that was importantly affecting his functioning. Based on patient 1, our findings show that a combination of (i) early diagnosis in the asymptomatic phase of HT1, (ii) regular monitoring of dietary protein intake and (iii) optimal metabolic control of tyrosine, phenylalanine, and SA can result in a favorable cognitive outcome. Non-favorable neuropsychological state at patient 2 was most likely caused by hyperammonemia, unmanageable coagulopathy and severe cerebral edema before liver transplantation.

The main study limitations are a retrospective design and a low number of patients presented. Furthermore, different genetics, disease courses and therapeutic approaches impede the direct comparison between both patients; however, our study can complement other multi-center studies with additional data on a rare disease, such as HT1.

## 4 Conclusions

Management of patients with HT1 is complex and requires a multidisciplinary team. NBS can assist clinicians in a timely diagnosis and treatment of HT1 and could possibly prevented an acute liver failure at one of the presented patients. In addition to NBS, retrograde diagnosis of HT1 from DBS is technically possible more than three years after birth. SA showed to be more reliable than tyrosine. Favorable cognitive outcome can be achieved by optimal metabolic control of tyrosine, phenylalanine and SA as demonstrated at patient 1. DBS represents a convenient sample for frequent amino acid monitoring. When clinical presentation and biochemical markers are characteristic of HT1 and an initial genetic analysis of the coding regions of the *FAH* gene does not confirm the diagnosis, further investigation of intronic (non-coding) regions is warranted.

## Compliance with ethics guidelines

All procedures followed were in accordance with the ethical standards of the responsible committee on human experimentation (institutional and national) and with the 2000 revision of Helsinki declaration. Written informed consent was obtained from the patient's parents for being included in the study and for anonymized data publication and are available for review upon request. This article does not contain any studies with animal subjects performed by any of the authors.

## Author contributions – CrediT author statement

*Jaka Sikonja:* Investigation, Formal analysis, Writing - Original Draft; *Jernej Brecelj:* Investigation, Formal analysis, Writing - Original Draft; *Mojca Zerjav Tansek:* Formal analysis, Writing - Review & Editing; *Barbka Repic Lampret:* Investigation, Writing - Review & Editing; *Ana Drole Torkar:* Investigation, Writing - Original Draft; *Simona Klemencic:* Investigation, Writing - Original Draft; *Neza Lipovec:* Investigation, Writing - Original Draft; *Valentina Stefanova Kralj:* Investigation, Writing - Original Draft; *Sara Bertok:* Investigation, Writing - Original Draft; *Jernej Kovac:* Investigation, Writing - Original Draft; *Barbara Faganel Kotnik:* Investigation, Writing - Original Draft; *Marketa Tesarova:* Investigation, Methodology, Resources; *Ziga Iztok Remec:* Investigation, Writing - Original Draft; *Marusa Debeljak:* Methodology, Writing - Review & Editing, *Tadej Battelino:* Conceptualization, Writing - Review & Editing, Supervision; and *Urh Groselj:* Investigation, Conceptualization, Formal analysis, Writing - Review & Editing.

## Declaration of Competing Interest

Authors declare no conflicts of interest.
